# Perceived Value of Information Attributes: Accounting for Consumer Heterogeneous Preference and Valuation for Traceable Agri-Food

**DOI:** 10.3390/foods12040711

**Published:** 2023-02-06

**Authors:** Ruifeng Liu, Jian Wang, Jiahao Liang, Hengyun Ma, Fei Liang

**Affiliations:** College of Economics and Management, Henan Agricultural University, Zhengzhou 450046, China

**Keywords:** traceable agri-food, heterogeneous preference, choice experiments, willingness to pay

## Abstract

Information attributes characterize traceable agri-food. The perceived value of information attributes influences consumers’ preferences for traceable agri-food, consisting of two dimensions, predictive value and confidence value. We examine heterogeneous preferences and willingness to pay (WTP) in China’s traceable agri-food market. Using the choice experiments, we explore how the traceability information, certification type, region of origin, and price influence Chinese consumers’ Fuji apple choices. We identify three consumer classes by a latent class model: certification-oriented class (65.8%), price-sensitive and origin-oriented class (15.0%), and no-buy class (19.2%). The results show that consumer sociodemographic characteristics, predictive value, and confidence value are the heterogeneous sources that determine their preferences for Fuji apple information attributes. Specifically, consumers’ age, family income per month, and whether the family has children under 18 significantly impact the membership probability of consumers in both certification-oriented and price-sensitive and origin-oriented classes. Consumers’ predicted value and confidence value significantly impact the membership probability of consumers in the certification-oriented class. In contrast, consumers’ predicted value and confidence value have no significant impact on the membership probability of consumers in price-sensitive and origin-oriented class.

## 1. Introduction

Increasing concerns about agri-food quality and safety have heightened the need for relevant business practices, industry strategies and policy initiatives. The traceability of food products is believed to be effective in reducing information asymmetries in quality and safety attributes [1]. To reduce uncertainty and facilitate market transactions, countries have implemented agri-food traceability systems to provide consumers with more food information [2]. As early as 2004, the State Council of China issued the decision of “Further Strengthening Food Safety Work”, which clearly initiated the establishment of a traceability system for agricultural products. Since 2007, a series of food safety scandals, particularly melamine-tainted infant formula, have accelerated the development of agri-food traceability systems. In 2010, China’s Ministry of Commerce introduced and funded a meat and vegetable circulation traceability system in 10 capable pilot cities [3]. Later, China launched voluntary traceability programs for apple, tea, pork, fresh milk, flounder, and other products in different regions. In recent years, China has taken substantial steps to improve the requirements and application of the traceability of food products [4,5,6].

One interesting phenomena is the high level of consumer concern about agri-food quality and safety [7] but low awareness and demand for traceable agri-food despite the growing interest in traceability from public policy and the private sector [8,9,10]. Information economics argues that traceability systems play an important role in communicating with consumers and reducing information asymmetries. Thus, there seems to be an assumption that consumers can benefit from traceable information and want more [2]. In addition, modern technology has helped to reduce the cost of information collection and processing, thereby increasing the utility of information to consumers. However, compared with other quality and safety information, traceability information does not attract more attention and pays a premium from consumers [11,12]. Although the traceability system is helpful to improve the ability of information transmission quality and safety information, irrelevant or useless information will lead to utility loss for consumers and increase time cost and cognitive pressure [13]. Therefore, it becomes important to evaluate traceable agri-food information. In particular, it becomes important to examine whether quality and safety information choices for traceable agricultural products are related to preference segmentation and identify potential determinants of their membership probabilities.

Previous research has examined consumer decisions about food traceability [14,15,16,17]. However, these studies failed to account for traceable food diversity. In addition, traceable food is treated as one-dimensional in quality attributes [18]. However, Hobbs et al. [2] differentiated the traceable agri-food in multiple dimensions of information attributes. Many studies have examined which information attributes consumers care more about to judge the quality and safety of traceable food when making a purchase decision [13,19,20], including traceability information, quality and safety assurance, the country of origin, animal welfare, worker safety, and environmental impact [12,21,22]. Although there is a distinction between ex ante quality assurance information and ex post reactive traceability information, the assessment of information attributes varies considerably in these literatures [23,24]. Some studies have shown that consumers are willing to pay more for traceable information [25,26,27]. However, other studies have shown that consumers are highly inclined to pay for quality assurance information [11,28].

The apple is the primary fruit produced and consumed in China. In 2019, the apple area and output accounted for 16.12 percent and 15.49 percent of China’s total fruit area and production, respectively (China Rural Statistical Yearbook 2020). China’s per capita fruit consumption was 51.4 kg (China Statistical Yearbook 2020), of which apples accounted for about two-thirds (http://finance.sina.com.cn/money/future/agri/2018-11-19/doc-ihmutuec1603058.shtml, (accessed on 6 July 2021)). Since 2014, however, China’s State Administration for Market Regulation has published the results of sample food safety tests online. The results showed that the residue of dichlorvos, chlorpyrifos, phosphorescence, didiazole, and other pesticides led to unqualified apple test results, even more than 30 times (https://spcjsac.gsxt.gov.cn/, (accessed on 9 July 2021)). If there is a problem with the quality and safety of apples, it will seriously threaten consumers’ health, curb market demand, and destroy the apple industry.

This study aims to determine the impact of consumers’ predictive value and confidence value on their heterogeneous preferences for information attributes of traceable Fuji apple products. Specifically, a choice experiment is applied to collect data on Chinese consumers’ perceived value and the evaluation of information attributes in six cities. A Latent Class (LC) model is used to estimate consumers’ preference and its heterogeneity, characterized by distinctive classes of the utility of information attributes of traceable Fuji apple products. Both Multinomial Logit (MNL) and Mixed Logit (MIXL) can model consumer heterogeneity; some researchers have compared the traceable food choices of these two models and found that the MIXL model performs better than the MNL model in traceable food choice survey datasets [29] because the MIXL model assumes not only heterogeneous preferences for unobserved attributes of traceable food but also extends heterogeneous preferences for observable attributes of traceable food [30]. In addition, to determine the impact of individual characteristics on heterogeneity, some studies have interacted individual characteristics with various attributes of traceable foods to generate new variables used to calculate their marginal utility [31,32]. However, LC models specify discrete distributions of heterogeneous tastes and can simultaneously predict traceable food choices and segment-specific members [33].

The contribution of this paper is twofold. First, studying traceable fruit will help expand the research and application field of the preference for traceable agricultural products. Second, this study uses consumers’ perception variables (i.e., the predictive value and confidence value) as well as socio-demographics to identify the source of consumers’ preference for traceable Fuji apple. Furthermore, it reveals the characteristics of different preference groups and willingness to pay for traceable apples, which can provide a more effective decision-making reference for the information attribute of traceable apples and market segmentation.

## 2. Materials and Methods

### 2.1. Attribute Specification

Following Liu et al. [10,34,35], we selected four information attributes to describe the different types of traceable Fuji apples, including traceability information, certification type, region of origin, and price. Traceability information is one of the primary information attributes of traceable apples. In this study, apple traceability information is designated into four levels: no traceability information (Notrace), traceability information in the production stage (Lotrace), traceability information in the production and processing stage (Mitrace), and traceability information in the production, processing, and distribution stage (Hitrace). Certification is also a key information attribute that consumers use to infer product quality and safety. There are four types of certification depending on the accrediting party: no certification (Nothcert), government certification (Govcert), domestic third-party certification (Dothcert), and international third-party certification (Inthcert). Region of origin is another information attribute of particular importance in fruit quality and safety evaluation [36]. In this study, Shandong, Shaanxi, and Xinjiang, three dominant apple producing areas in China, were selected as the producing areas of the choice experiments.

In this study, the price attribute of Fuji apple includes four levels: 6 yuan per 500 g, 8 yuan per 500 g, 10 yuan per 500 g, and 12 yuan per 500 g. The setting of the price attribute is the key to measuring the value of the information attribute. According to the field survey of supermarkets, markets, fruit shops, and farmers’ markets, the average market price of ordinary Fuji apples in the surveyed cities is about 6 yuan per 500 g. Therefore, we added 2 yuan per 500 g per level to the base price level of 6 yuan per 500 g to generate three additional price levels. The markup was based on discussions with apple sellers in China, and WTP estimates from previous research by Jin et al. [5]. Table 1 presents the information attributes and levels of settings for the traceable Fuji apple in this study.

### 2.2. Experimental Design

**Figure 1:**
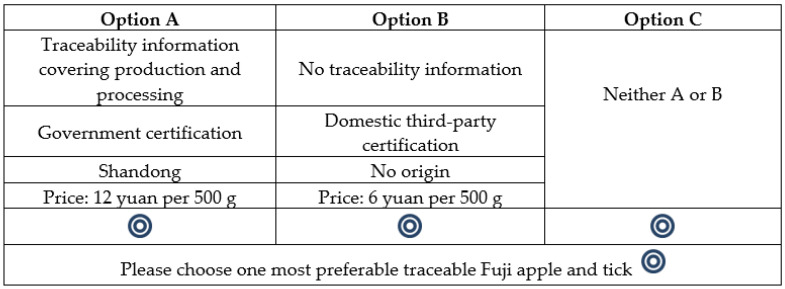
A sample choice task in the choice experiments.

An optimal selection of experimental designs should be statistically and cognitively valid [37]. Based on the four selected attributes and levels, the full factorial design can create 256 possible Fuji apple profiles (4 × 4 × 4 × 4). Next, two 256 Fuji apple profiles are randomly paired to construct 65,280 choice sets (256 × 255 = 65,280). While the full factorial design ensured that all possible attribute effects could be estimated independently, participants could not evaluate all choice sets regarding cognitive efficiency. Therefore, we used fractional factorial design to select subsets from the full factorial. This design improves cognitive efficiency, but the presence of subset alternatives may not be orthogonal and balanced. To balance the trade-off between cognitive and statistical efficiency, we used a fractional factorial design that maximizes the D-efficiency of the design matrix and minimizes the number of choice sets [38,39]. The statistical results of D-efficiency of the final choice experiments are shown in Table A1 of Appendix A. The 120 choice sets were randomly divided into ten versions with each participant answering only 12 choice sets. In addition, participants were given two Fuji apple products’ options, each with a “no-buy” option. Including a “no-buy” option makes a choice set more realistic because consumers will decide not to buy anything if they are unsatisfied with the available product options. In addition, the sequential effect of preference learning is mitigated by processing the repetitive choice task [40,41]. Finally, the choice set was presented randomly, suggested by [42]. An example choice set is provided in Figure 1.

### 2.3. Data Collection

From July to October 2017, we conducted face-to-face interviews in Beijing, Shanghai, Guangzhou, Xi’an, Harbin, and Jinan to obtain the data for this study. We chose convenient samples in these six cities for two main reasons: first, they represent the types of cities with unique cultural, economic, and geographical characteristics in China. Second, sample consumers need to understand traceable agricultural products thoroughly to improve the experiment’s effectiveness. At present, traceable agricultural products are mainly sold in cities. In addition, these six cities have national or provincial food traceability system construction, so the sample cities have the research conditions of preference for traceable agricultural products. We conveniently intercepted participants in sample cities at supermarkets, farmer markets, and fruit stores. Each participant starts by selecting a choice task. To reduce the bias of the hypothesis, subjects were shown a “cheap talk” script (see Appendix B) before the choice experiments [43,44,45,46]. Participants were then asked to answer a follow-up questionnaire. The questionnaire collected information about the participants’ demographics, the perceived value of traceability information, types of certification, and region of origin. The investigation took about 15 min.

### 2.4. Econometric Modelling

To account for preference heterogeneity among potential customers with different characteristics, especially the perceived value of information attributes, we use the LC model based on stochastic utility theory [47]. For consumer n, the utility from choosing alternative i in choice scenario t is given by:

(1) $$U_{nit}=\beta x_{nit}+\varepsilon_{nit}$$

where $x_{nit}$ is a vector of information attributes of alternative i. Specifically, $x_{nit}$ includes $Price_{nit}$, which is a continuous variable represented by the price level of the four experimental designs; $LOTRACE_{nit}$, $MITRACE_{nit}$, and $HITRACE_{nit}$, indicating the categorical variables of traceability information of Fuji apple products where no traceability information is the base category; $GOVERT_{nit}$, $DOTHCERT_{nit}$, and $INTHCERT_{nit}$, indicating the categorical variables of certification type of Fuji apple products, respectively, where no certification is the base category; and $XJ_{nit}$, $SD_{nit}$, and $SHX_{nit}$, where are the categorical variables of Fuji apple products’ region of origin claim. β is a vector of the utility coefficients of each information attribute above. They are the non-price attribute coefficients, which are assumed to be random following a normal distribution. $\varepsilon_{nit}$ is a random component that is not included in deterministic utility $\beta x_{nit}$. When $\varepsilon_{nit}$ is following a Type I extreme value distribution (assuming *iid*), the Conditional Logit (CL) model can be used to estimate the probability of consumer n choosing alternative i in the choice scenario t. The probability can be expressed as:

(2) $$P_{nit}=\frac{exp\left(\beta x_{nit}\right)}{\sum_{j=1}^{J}exp\left(\beta x_{njt}\right)}$$

According to the experimental design, the joint probability of consumer n choosing alternative i among the sequence of 12 choice scenarios is:

(3) $$L_{ni}=\prod_{t=1}^{12}\frac{exp\left(\beta x_{nit}\right)}{\sum_{j=1}^{J}exp\left(\beta x_{njt}\right)}$$

where β is homogeneous assuming that consumers’ tastes are homogeneous. If this restrictive assumption is relaxed, the weighted average value of the logit formula under different β values is given by the density $f\left(\beta \right)$, which is the mixed logit probability [48].

(4) $$P_{ni}=\int L_{ni}\left(\beta \right)f\left(\beta \right)d\beta$$

Equation (4) is a flexible combination of choice probability models based on random utility theory [49]. When $f\left(\beta \right)$ is assumed to be continuously distributed, such as a normal or lognormal distribution, most studies often refer to the choice probability as a mixed logit. When $f\left(\beta \right)$ is specified to be discrete with finite values of β, the choice probability becomes the LC model. The LC model can generate a limited number of consumers and capture the preference heterogeneity among different consumer groups. However, it is unknown from the data which category consumers fall into [50]. Therefore, assuming that there are C different classes and consumer n is in class c, the probability of choosing alternative i can be expressed as:

(5) $$P_{ni}\left(\beta_{c}\right)=\sum_{c=1}^{C}L_{ni}\left(\beta_{c}\right)H_{nc}$$

where $\beta_{c}$ and $L_{ni}\left(\beta_{c}\right)$ are the utility parameter vector and the joint probability vector of choosing alternative i for a given class c, respectively. $H_{nc}$ is the prior class probability of consumer n, which can be defined as:

(6) $$H_{nc}=\frac{exp\left(\theta_{c}z_{n}\right)}{\sum_{r=1}^{c}exp\left(\theta_{r}z_{n}\right)}$$

where $z_{n}$ is the eigenvector of consumer n, and $\theta_{c}$ is the class c covariates parameter vector. Then, $\theta_{c}$ and $\beta_{c}$ can be estimated by the maximum likelihood method. These two estimates ${\hat{\theta }}_{c}$ and ${\hat{\beta }}_{c}$ are used to calculate ${\hat{H}}_{nc}$ and ${\hat{L}}_{ni}\left({\hat{\beta }}_{c}\right)$. Finally, from the Bayesian perspective, the posterior probability that consumer choosing alternative i belongs to class c can be written as:

(7) $${\hat{H}}_{c|i}=\frac{{\hat{H}}_{nc}\left({\hat{\theta }}_{c}\right){\hat{L}}_{ni}\left({\hat{\beta }}_{c}\right)}{\sum_{c=1}^{C}{\hat{H}}_{nc}{\hat{\theta }}_{c}{\hat{L}}_{ni}\left({\hat{\beta }}_{c}\right)}\;$$

Thus, we can estimate the selected parameter and the segment membership probability to account for the heterogeneous preference for traceable Fuji apples [51].

In addition, the WTP for Fuji apple attribute is calculated by $\mathrm{WTP}=-\frac{\beta_{k}}{\beta_{p}}$, where $\beta_{k}$ is the coefficient of non-price attribute k, and $\beta_{p}$ is the estimated price coefficient. Dummy coding was used for the non-price attributes. Moreover, 95% confidence intervals were estimated using the parametric bootstrapping procedure suggested by [52].

## 3. Results

### 3.1. Descriptive Analysis

Table 2 shows the participants’ sociodemographic characteristics in the sample cities. Most of the samples’ demographics are consistent with the overall demographics of the six cities surveyed and China, indicating that the sample in our study is representative of the target population (see Table A2 in Appendix C). In this study, about half of the respondents were male, accounting for 50.72%. About 36.33% of the samples were between 25 and 34 years old, and about 57.41% had 13 to 16 years of education. The monthly household income of most participants ranged from 10,000 to 19,999 yuan. In addition, about 44.74% of the sample households had children under 18.

Table 2 also reports the consumers’ perception of predictive value and confidence value of attributes. The predictive value of each attribute was derived from three questions, respectively: “Do you think traceability information can predict food quality and safety?”, “Do you think certification information can predict food quality and safety?”, and “Do you think region of origin information can predict food quality and safety?”. The answer was “1 = no; 2 = uncertain; 3 = yes”, respectively. The confidence value was also derived from three questions, respectively: “Are you sure the apples you bought are traceable?”, “Are you sure the apples you bought are certified?”, and “Are you sure the apples you bought are originated from where?”. The answer was “0 = no; 1 = yes”, respectively. Regarding predictive value, about 44.6%, 26.72%, and 38.1% of the sample participants believed that traceability, certification, and region of origin information can ensure the quality and safety of apple, respectively. In terms of confidence value, about 65.44%, 53.78%, and 45.79% of the samples could identify the traceability, region of origin, and certification information, respectively.

### 3.2. Choosing the Number of Classes

We run a canonical search to explore the optimal number of segments of participants’ preference heterogeneity between two and ten classes. The performance of latent class models is usually compared by examining three information criteria: ρ^2^, Akaike information criterion (AIC), and minimum Bayesian information criterion (BIC) [53,54]. If the LC logit model best fits the data, ρ^2^ should be maximized and AIC and BIC minimized. However, in our study, ρ^2^ increased from 0.12 to 0.18 while AIC and BIC decreased throughout the process (see Table 3). Following Boxall and Adamowicz [51] and Thiene et al. [55], when adding a class, the optimal solution is selected based on the marginal improvement of the criterion value. Because of all the values of information criteria (i.e., ρ^2^, AIC and BIC) in the data increased significantly, we chose three classes.

### 3.3. Heterogeneous Preference

To further identify preference heterogeneity, we compared the results of CL and MIXL models in Table 4. We specified normal distribution parameters for non-price attributes in the MIXL model after testing various distributions. In addition, it is assumed that the coefficients and prices of ASC (Chooseno variable) are fixed. As shown in Table 4, the coefficient on the no-buy option (Chooseno) was negative and significant in the MIXL model, meaning that the utility of not choosing either option was less than the utility of choosing any of the proposed product alternatives set. As expected, the price coefficient was negative and significant, indicating that price increased reduce utility. The estimated coefficients for all attributes of Fuji apple were positive and significant at the 1% level. This means that all of these attributes significantly influenced consumers’ preference for Fuji apples. Moreover, the results of the MIXL model indicate strong heterogeneity in consumer preferences for Fuji apple attributes, as the estimated standard deviations of all attributes were significantly different from zero.

### 3.4. Characterizing of Class Preference and WTP

Table 5 reports the estimated of parameters for the three-class model. In terms of the probability of membership in the preferred classes, our results showed that the probability of respondents belonging to class one, class two, and class three was 65.8%, 15.0%, and 19.2%, respectively. We observed that consumer sociodemographic characteristics, the predictive value and confidence value were significant in predicting class membership.

Fuji apple’s traceability information, certification, and region of origin significantly and positively impacted the respondents in class one. Consumers valued certification types more than traceability information and region of origin. Furthermore, government certification (1.405) was rated as the most important type of certification, followed by international third-party certification (1.295) and domestic third-party certification (1.139). Therefore, we named class one “certification-oriented”. Respondents in the class one were more likely to choose Fuji apples, which cost less. Furthermore, respondents in class one were younger, more educated, and had children under 18 years of age in their households. We noted that the consumer household monthly income variable was statistically significant in class one but not in the economic sense. Consumers in class one may have higher perceptions of “Traceability and certification information can predict food quality and safety, respectively” than class three consumers. In addition, consumers in class one may have higher confidence in identifying the certification and region of origin of Fuji apple than those in class three.

In our study, consumers in class two preferred region of origin attributes to class three. In addition, the Shandong Fuji apple ranked first in all region of origin attributes (1.302). This indicates that class two respondents had a significantly higher probability of choosing Fuji apples associated with region of origin. In addition, a key feature of class two was the higher price parameter value, indicating that class two had a higher price sensitivity than class three. Therefore, we named class two “price-sensitive and origin-oriented”. Sample consumers in class two were younger, had children in the household, and had more family income per month than respondents in class three. Moreover, as with class one consumers, the household monthly income variable was statistically significant in class two but not economically significant. However, there was no significant difference in consumers’ perceptions of “Traceability, certification and region of origin information can predict food quality and safety, respectively” between class two and class three. In addition, all the consumer confidence value variables in class two were insignificant compared to class three. That is, there was no significant difference in consumers’ confidence in identifying “Are you sure the apples you bought are traceable, certified, and originated from where, respectively?”.

In addition, the coefficient of Chooseno variable in class three was positive and significant, and its value was larger than the coefficient estimate value of other Fuji apple attributes, indicating that the class three consumer tended not to choose the Fuji apple. Therefore, we labeled it as “no-buy”.

Table 6 reports WTP estimates for three classes in this study. The results showed that the marginal WTP of Fuji apple varied significantly between different food attributes and consumer classes. We found that the premium paid by consumers in certification-oriented class for Fuji apple attributes was significantly higher than that of the other three categories. Consumers from no-buy class paid a significantly lower premium for each attribute of Fuji apple than the other classes.

## 4. Discussion

This study shed light on Chinese consumers being very concerned about agricultural products’ quality and safety. However, the cognition and utilization rate of agricultural product quality and safety traceability were low. In line with Wu et al. [56] and Liu et al. [35], this study found that Chinese consumers have low confidence in and satisfaction with a traceable food system that ensures the quality and safety of agricultural products. In addition, consumers’ trust in traceability information was an important factor affecting their perception of the value of agricultural product traceability information. This finding is consistent with Garaus and Treiblmaier [57].

It is important to determine whether complete traceability information is valuable to producers and can induce consumer preferences and price premiums. In our study, we found that Chinese consumers are willing to pay a premium for traceability information necessary to operate agricultural product quality and safety traceability systems effectively. This finding is consistent with Shew et al. [27]. Furthermore, based on the heterogeneity of consumer preferences, the market value of traceable agrarian products can be realized only by providing a portfolio of traceable information that meets the market demand. This is the similar argument as Verbeke and Roosen [58], Wongprawmas and Canavari [59], and El Benni et al. [60].

Certification information with its independence and authority to convey product quality and safety information to consumers [61,62]. Consumers prefer a particular certification when they believe there seems to be a match between the certificate and their quality assurance ability. In this study, we found that certification has a significant impact on consumers’ preference and WTP. The results are in line with Lusk et al. [63] and Carter and Cachelin [64]. In addition, we suggested the certification may be the source of consumers’ heterogeneous preference for Fuji apple products. Meanwhile, Chen et al. [65] and Brach et al. [66] share the similar view.

The region of origin is not simply a marker used to trace the source of information. Specifically, the origin is a stimulus for information on fruit quality and safety, as fruit quality is not only related to the institutional and regulatory environment of the region, but is also highly related to the region’s natural environment [67]. Similarly, we found that region of origin has a significant impact on consumers’ preference and WTP. In addition, we suggested region of origin was an important source of product evaluation and heterogeneous preferences, as are brand signals of quality and safety levels. These findings are consistent with those of Kerr [68], Darby et al. [69], and Ortega et al. [70].

Some studies have analyzed preference heterogeneity by combining sociodemographic factors [69,71,72]. Some other studies include psychological factors, such as attitudes and perceptions about traceable food, to account for heterogeneity [73,74,75]. In the psychology literature, consumer behavior, and marketing, Cox [76] developed models based on predictive and confidence value (Predictive value refers to the strength of the relationship consumers perceive or believe between some item of information (i.e., the generic cue) and product quality. Confidence value means the degree to which consumers have self-confidence in their ability to distinguish, evaluate and judge some item of information accurately). In this study, we found that predictive value and confidence value, and consumers’ individual-specific characteristics (i.e., consumers’ age, family income per month, education level, and whether the family has children under 18) can explain the heterogeneity of information preference. Furthermore, Grunert [77] and Halawany et al. [78] found that, although predictive value and confidence value are both necessary for information utilization in the quality perception process, empirical evidence demonstrates that confidence value is the basis for dealing with information.

The study has three limitations. First, to understand consumers’ demands for information traceability, considering that too many attributes may cause experimental bias, this study only set the attribute level of traceability information from the dimension of traceability depth (Golan et al. [79] defined a traceability system as an information system that tracks certain products or product characteristics in the supply chain. They set three criteria to measure traceability systems: breadth, depth, and precision. The breadth is the range of information recorded by the traceability system. Depth is the length that can be traced backward or forward. Accuracy is the ability to determine the source of a problem or a feature of a product). If consumer preference for traceability breadth information can be studied at the same time (for example, the information preference of pesticide residues, fertilizer application, and heavy metal pollution in the planting process), it will make the research on traceability information more comprehensive and systematic. Future research could include other informational attributes of apples, such as organic certification, as well as other attributes, such as freshness, appearance, and taste. Second, we used the widely recognized “cheap talk” method to build a more realistic product profile and selection scenario. However, the estimated results were subject to a bias as other stated preferences. Therefore, we may consider using explicit preference methods, such as auctions or natural selection experiments, to solve this problem. Third, the samples of this study were from first-tier cities and provincial capitals in China. In the future, different samples of urban and even rural consumers may be considered to draw more comprehensive conclusions.

## 5. Conclusions and Policy Implications

First, our results showed that consumer evaluations of Fuji apple product information attributes varied depending on the attribute type. In this study, the Fuji apple products’ information attribute most valued by consumers was the certification type, followed by region of origin and traceability information. Second, we identified three latent classes, i.e., certification-oriented class (65.8% in market share), price-sensitive and origin-oriented class (15.0% in market share), and no-buy class (19.2% in market share). Third, we found that consumers’ socio-demographic characteristics and predictive value and confidence value are heterogeneous sources that influence consumers’ preference for information attributes of Fuji apple products.

These findings provide important policy implications for producers and marketers in China’s fresh fruit industry. Our research shows that consumers were willing to pay different premiums for Fuji apple products with different information levels. As a result, producers and operators can use labels with different traceability features to distinguish them from competitors. Given the high evaluation of food-certified Fuji apple products by Chinese consumers, by improving the regulatory environment, cultivating the integrity awareness of market producers and operators, strengthening the publicity of traceable agricultural products, and further improving the predictive value of information and consumers’ confidence in traceable agrarian products, agricultural products can gain more consumer recognition and trust, and thus enhance their market competitiveness.

## Figures and Tables

**Table 1:** Fuji apple information attributes in the choice experiment.

Information Attribute	Level	Description
Traceability information	4	Traceability information of production, processing and distribution stages in the apple supply chain (Hitrace)
		Traceability information of production and processing stages in the apple supply chain (Mitrace)
		Traceability information including production stage in the apple supply chain (Lotrace)
		No traceability information (Notrace)
Certification type	4	Government certification (Govcert)
		Domestic third-party certification (Dothcert)
		International third-party certification (Inthcert)
		No certification (Nothcert)
Region of origin	4	Produced in Shandong (Shandong)
		Produced in Xinjiang (Xinjiang)
		Produced in Shaanxi (Shaaxi)
		No region of origin (Noorigin)
Price	4	12 yuan per 500 g
		10 yuan per 500 g
		8 yuan per 500 g
		6 yuan per 500 g

Note: In July 2017, 1 US dollar = 6.77 yuan.

**Table 2:** Socio-demographic characteristics and perceived value of Fuji apple products’ information attribute of the sample.

Variables	Definition	Numbers	%
Gender	Male	1061	50.72
	Female	1031	49.28
Age (years)	≤24	533	25.48
	25~34	760	36.33
	35~44	375	17.93
	45~54	216	10.33
	55~64	138	6.60
	≥65	70	3.35
Education level (years)	≤9	243	11.62
	10~12	333	15.92
	13~16	1201	57.41
	>16	315	15.06
Monthly family income (yuan)	<5000	157	7.50
	5000~9999	535	25.57
	10,000~19,999	773	36.95
	20,000~29,999	338	16.16
	30,000~39,999	138	6.60
	40,000~49,999	44	2.10
	50,000~59,999	47	2.25
	60,000~99,999	30	1.43
	≥100,000	30	1.43
Whether the family has children under 18	1 = Yes, 0 = No	936	44.74
Predictive value of traceability	No	175	8.37
	Uncertain	984	47.04
	Yes	933	44.60
Predictive value of certification	No	146	6.98
	Uncertain	1387	66.30
	Yes	559	26.72
Predictive value of region of origin	No	723	34.56
	Uncertain	572	27.34
	Yes	797	38.10
Confidence value of traceability	No	723	34.56
	Yes	1369	65.44
Confidence value of certification	No	1134	54.21
	Yes	958	45.79
Confidence value of region of origin	No	967	46.22
	Yes	1125	53.78

**Table 3:** Comparison of LC model with different number of classes.

No. of Classes	No. of Parameters (P)	AIC	ρ^2^	BIC	LL	LL (0)
2	23	39,228.20	0.12	39,358.05	−19,591.10	−22,303.03
3	35	38,662.20	0.13	38,859.81	−19,296.10	−22,238.90
4	47	37,939.55	0.15	38,204.91	−18,922.78	−22,268.04
5	59	37,611.99	0.15	37,945.10	−18,746.99	−22,244.19
6	71	37,036.72	0.16	37,437.57	−18,447.36	−22,174.04
7	83	36,965.05	0.17	37,433.66	−18,399.53	−22,202.84
8	95	36,719.58	0.17	37,255.94	−18,264.79	−22,196.35
9	107	36,536.79	0.18	37,140.90	−18,161.39	−22,177.16
10	119	36,469.73	0.18	37,141.59	−18,115.87	−22,125.46

Notes: AIC is computed using the formula AIC = −2LL + 2P. ρ^2^ is computed using the formula ρ^2^ = 1 − AIC/[−2LL (0)]. BIC = −2LL + P × ln (N). LL is Log Likelihood at Convergence. LL (0) is Log Likelihood at 0. N is the total number of 75,312 choices from 2092 respondents.

**Table 4:** Estimates from Conditional Logit model (CL model) and Mixed Logit model (MIXL model).

Attributes	CL Model	MIXL Model	
	Mean	Mean	Standard Deviation
Price	−0.165 *** (0.004)	−0.248 *** (0.010)	−
Chooseno	−0.379 *** (0.052)	−0.597 *** (0.106)	−
Traceability:			
Hitrace	0.825 *** (0.029)	1.159 *** (0.051)	0.898 *** (0.070)
Mitrace	0.632 *** (0.027)	0.900 *** (0.046)	0.894 *** (0.062)
Lotrace	0.407 *** (0.026)	0.573 *** (0.038)	−0.401 *** (0.089)
Certification:			
Govcert	1.165 *** (0.028)	1.594 *** (0.055)	1.338 *** (0.061)
Dothcert	0.938 *** (0.028)	1.261 *** (0.048)	0.907 *** (0.061)
Inthcert	1.059 *** (0.028)	1.456 *** (0.053)	1.257 *** (0.060)
Region of Origin:			
Xinjiang	0.898 *** (0.028)	1.185 *** (0.050)	1.194 *** (0.063)
Shandong	0.943 *** (0.029)	1.261 *** (0.052)	1.237 *** (0.058)
Shaanxi	0.932 *** (0.028)	1.203 *** (0.047)	1.186 *** (0.061)
Log Likelihood	−22,307.07	−20,700.08	
LR chi2	10,544.99	−	
Ward chi2	−	2802.79	
Pseudo R2	0.191	−	
Prob > chi2	−	0.0000	
Observations	75,312	75,312	

Notes: The attribute names of the Fuji apple products are shown in Table 1. Standard errors in parentheses. *** represents the significance level of 1%.

**Table 5:** LC model with perceived value and confidence value as class membership.

Variable	Certification-Oriented (Class 1)	Price-Sensitive & Origin-Oriented (Class 2)	No-Buy (Class 3)
Class membership	0.658	0.150	0.192
Attributes:			
Price	−0.087 *** (0.007)	−0.120 *** (0.019)	−0.711 *** (0.039)
Chooseno	−0.950 *** (0.099)	2.375 *** (0.325)	−4.963 *** (0.403)
Lotrace	0.515 *** (0.033)	0.321 *** (0.113)	0.241 ** (0.102)
Mitrace	0.825 *** (0.036)	0.591 *** (0.114)	0.215 ** (0.107)
Hitrace	1.026 *** (0.039)	0.917 *** (0.115)	0.384 *** (0.113)
Govcert	1.405 *** (0.039)	1.208 *** (0.133)	0.623 *** (0.115)
Dothcert	1.139 *** (0.037)	0.790 *** (0.131)	0.520 *** (0.102)
Inthcert	1.295 *** (0.038)	0.882 *** (0.126)	0.748 *** (0.097)
Xinjiang	1.063 *** (0.037)	1.100 *** (0.146)	1.069 *** (0.106)
Shandong	1.102 *** (0.038)	1.302 *** (0.138)	0.980 *** (0.105)
Shaanxi	1.009 *** (0.038)	1.052 *** (0.150)	1.436 *** (0.108)
Class membership			
Gender	0.122 (0.137)	−0.143 (0.181)	−
Age	−0.024 *** (0.005)	−0.018 ** (0.008)	−
Family income per month	0.000 ** (7.46 × 10^−6^)	0.000 ** (7.66 × 10^−6^)	−
Education	0.068 *** (0.018)	0.029 (0.030)	−
Child	0.365 *** (0.138)	0.355 ** (0.181)	−
Predictive value:			
Predictive value of traceability	0.210 * (0.114)	−0.174 (0.213)	−
Predictive value of certification	0.251 * (0.133)	−0.170 (0.262)	−
Predictive value of region of origin	−0.047 (0.137)	−0.177 (0.184)	−
Confidence value:			
Confidence value of traceability	−0.014 (0.148)	−0.069 (0.199)	−
Confidence value of certification	0.260 * (0.145)	−0.040 (0.195)	−
Confidence value of region of origin	0.175 ** (0.084)	0.073 (0.123)	−

Notes: Standard errors in parentheses. *, ** and *** represent the significance level of 10%, 5% and 1%, respectively.

**Table 6:** WTP estimates of each class.

Attributes	Certification-Oriented (Class 1)	Price-Sensitive & Origin-Oriented (Class 2)	No-Buy (Class 3)
Lotrace	5.95 [4.77, 7.13]	2.67 [0.52, 4.82]	0.34 [0.05, 0.63]
Mitrace	9.52 [7.79, 11.26]	4.91 [2.23, 7.58]	0.30 [−0.01, 0.61]
Hitrace	11.84 [9.78, 13.90]	7.61 [4.31, 10.92]	0.54 [0.20, 0.88]
Govcert	16.22 [13.49, 18.96]	10.03 [5.88, 14.18]	0.88 [0.51, 1.24]
Dothcert	13.15 [10.88, 15.41]	6.56 [3.42, 9.70]	0.73 [0.42, 1.04]
Inthcert	14.95 [12.38, 17.52]	7.32 [4.07, 10.57]	1.05 [0.74, 1.36]
Xinjiang	12.27 [10.12, 14.42]	9.14 [4.90, 13.37]	1.50 [1.15, 1.85]
Shandong	12.72 [10.52, 14.92]	10.81 [6.19, 15.44]	1.38 [1.03, 1.72]
Shaanxi	11.65 [9.64, 13.66]	8.74 [4.65, 12.82]	2.02 [1.63, 2.41]

Note: Numbers in brackets represent 95% confidence intervals.

## Data Availability

Not applicable.

## Appendix A

**Table A1:** A priori estimates of standard errors for attribute levels.

Attribute	Level	Frequency	Actual Standard Deviation	Ideal Standard Deviation	Efficiency
Traceability information	Lotrace	60	0.2270	0.2346	1.0679
	Mitrace	60	0.2293	0.2346	1.0468
	Hitrace	60	0.2496	0.2346	0.8839
	Notrace	60	-	-	-
Certification type	Govcert	61	0.2277	0.2325	1.0426
	Dothcert	59	0.2391	0.2325	0.9458
	Inthcert	60	0.2341	0.2325	0.9865
	Nocert	60	-	-	-
Region of origin	Shandong	61	0.2456	0.2325	0.8958
	Xinjiang	60	0.2386	0.2325	0.9496
	Shaanxi	60	0.2341	0.2325	0.9862
	Noorigin	59	-	-	-
Price	12 yuan per 500 g	59	0.2224	0.2294	1.0645
	10 yuan per 500 g	60	0.2411	0.2294	0.9055
	8 yuan per 500 g	60	0.2229	0.2294	1.0593
	6 yuan per 500 g	61	-	-	-

Notes: Task generation method is “Balanced Overlap” using a seed of 1. The efficiencies reported above for this design assume an equal number of respondents complete each version. Ideal standard deviation is the standard deviation that meets the orthogonality condition.

## Appendix B

### Cheap Talk Script

“We know from past studies that people often respond in one way but behave differently. For example, several people state a higher WTP than what one was willing to pay for the product in a grocery store. However, no one has to pay to show a particular preference. A possible reason for this is that people do not think about the finite amount of money we have. When you do not need to pay, generosity is easy. But when we’re in the grocery store, we have to spend money if we decide to buy this good. In any case, we ask you to answer the preferences and WTPs of each question, just like you have to pay for your choice in an actual grocery. Please keep this in mind when answering the last few questions.”

## Appendix C

**Table A2:** Comparison of socio-demographic characteristics between participants in % of the six cities.

Variable	Beijing	Shanghai	Guangzhou	Xi’an	Jinan	Harbin
Gender						
Male	51.21	49.61	50.16	50.49	49.66	50.26
Female	48.79	50.39	49.84	49.51	50.34	49.74
Age (years)						
≤18	13.1	11.61	17.97	14.13	16.4	13.9
18–60	71	56.82	64.27	75.51	70.4	66.1
>60	15.9	31.57	17.76	10.36	13.2	20
Education level (years)						
≤9	35.38	48.66	64.82	68.14	70.88	70.42
10–12	19.16	21.29	21.34	19.07	16.84	16.11
13–16	38.07	27.18	13.5	12.38	11.83	13.06
>16	7.39	2.87	0.34	0.5	0.45	0.41
Family size (No. of people)						
≤2	51.73	57.05	45.75	36.08	42.04	46.49
3	29.38	26.2	18.56	25.39	29.86	33.99
4	9.9	9.2	16.16	21.05	18.41	10.66
≥5	8.99	7.55	19.53	17.48	9.69	8.86

Notes: The data are from the National Bureau of Statistics of 2016. Average personal monthly income of Beijing is 4772.92 yuan, Shanghai is 4807.67 yuan, Guangzhou is 4616.67 yuan, Xi’an is 2969.17 yuan, Jinan is 3587.67 yuan, and Harbin is 2765.83 yuan, respectively.

## References

[1] Hobbs J.E. (2004). Information asymmetry and the role of traceability systems. Agribusiness.

[2] Hobbs J.E., Bailey D.V., Dickinson D.L., Haghiri M. (2005). Traceability in the Canadian red meat sector: Do consumers care?. Can. J. Agric. Econ..

[3] Charlebois S., Sterling B., Haratifar S., Naing S.K. (2014). Comparison of global food traceability regulations and requirements. Compr. Rev. Food Sci. Food Saf..

[4] Zhang X., Zhang J., Liu F., Fu Z., Mu W. (2010). Strengths and limitations on the operating mechanisms of traceability system in agro food, China. Food Control.

[5] Jin S., Zhang Y., Xu Y. (2017). Amount of information and the willingness of consumers to pay for food traceability in China. Food Control.

[6] Moruzzo R., Riccioli F., Boncinelli F., Zhang Z., Zhao J., Tang Y., Tinacci L., Massai T., Guidi A. (2020). Urban consumer trust and food certifications in China. Foods.

[7] Zhang J., Waldron S., Dong X. (2022). Evidence from a Choice Experiment in Consumer Preference towards Infant Milk Formula (IMF) in the Context of Dairy Revitalization and COVID-19 Pandemic. Foods.

[8] Wu L., Wang H., Zhu D. (2015). Analysis of consumer demand for traceable pork in China based on a real choice experiment. China Agric. Econ. Rev..

[9] Wu L., Wang H., Zhu D., Hu W., Wang S. (2016). Chinese consumers’ willingness to pay for pork traceability information—The case of Wuxi. Agric. Econ..

[10] Liu R., Gao Z., Snell H.A., Ma H. (2020). Food safety concerns and consumer preferences for food safety attributes: Evidence from China. Food Control.

[11] Cicia G., Colantuoni F. WTP for traceable meat attributes: A meta-analysis. Proceedings of the International European Forum on System Dynamics and Innovation in Food Networks.

[12] Lu J., Wu L.H., Wang S.X., Xu L.L. (2016). Consumer preference and demand for traceable food attributes: A choice-based conjoint analysis. Br. Food J..

[13] Jin S., Zhou L. (2014). Consumer interest in information provided by food traceability systems in Japan. Food Qual. Prefer..

[14] Lichtenberg L., Heidecke S.J., Becker T.C. Traceability of meat: Consumers’ associations and their willingness-to-pay. Proceedings of the Congress of European Association of Agricultural Economists.

[15] Ubilava D., Foster K. (2009). Quality certification vs. product traceability: Consumer preferences for informational attributes of pork in Georgia. Food Policy.

[16] Lee J.Y., Han D.B., Nayga Jr R.M., Lim S.S. (2011). Valuing traceability of imported beef in Korea: An experimental auction approach. Aust. J. Agric. Resour. Econ..

[17] Zheng S., Xu P., Wang Z., Song S. (2012). Willingness to pay for traceable pork: Evidence from Beijing, China. China Agric. Econ. Rev..

[18] Caswell J.A., Noelke C.M., Mojduszka E.M., Krissoff B., Bohman M., Caswell J.A. (2002). Unifying two frameworks for analyzing quality and quality assurance for food products. Global Food Trade and Consumer Demand for Quality.

[19] Mennecke B., Townsend A., Hayes D.J., Lonergan S.M. (2006). Study of the factors that influence consumer attitudes toward beef products using the conjoint market analysis tool. J. Anim. Sci..

[20] Verbeke W., Ward R.W. (2006). Consumer interest in information cues denoting quality, traceability and origin: An application of ordered probit models to beef labels. Food Qual. Prefer..

[21] Meuwissen M.P.M., Velthuis A.G.J., Hogeveen H., Huime R.B.M. (2003). Traceability and certification in meat supply chains. J. Agribus..

[22] Loureiro M.L., Umberger W.J. (2007). A choice experiment model for beef: What US consumer responses tell us about relative preferences for food safety, country-of-origin labeling and traceability. Food Policy.

[23] Dickinson D.L., Bailey D.V. (2002). Meat traceability: Are US consumers willing to pay for it?. J. Agric. Resour. Econ..

[24] Wu W., Zhang A.R., van Klinken R.D., Schrobback P., Muller J.M. (2021). Consumer trust in food and the food system: A critical review. Foods.

[25] Dickinson D.L., Bailey D.V. (2005). Experimental evidence on willingness to pay for red meat traceability in the United States, Canada, the United Kingdom, and Japan. J. Agric. Appl. Econ..

[26] Bai J., Zhang C., Jiang J. (2013). The role of certificate issuer on consumers’ willingness-to-pay for milk traceability in China. Agric. Econ..

[27] Shew A., Snell H., Nayga R.M., Lacity M. (2021). Consumer valuation of blockchain traceability for beef in the United States. Appl. Econ. Perspect. Policy.

[28] Yin S., Lv S., Chen Y., Wu L., Chen M., Yan J. (2018). Consumer preference for infant milk-based formula with select food safety information attributes: Evidence from a choice experiment in China. Can. J. Agric. Econ..

[29] Lagerkvist C.J., Amuakwa-Mensah F., Mensah J.T. (2018). How consumer confidence in food safety practices along the food supply chain determines food handling practices: Evidence from Ghana. Food Control.

[30] Fiebig D.G., Keane M.P., Louviere J.J., Wasi N. (2010). The generalized multinomial logit model: Accounting for scale and coefficient heterogeneity. Mark. Sci..

[31] Lim K.H., Hu W., Maynard L.J., Goddard E.W. Stated preference and perception analysis for traceable and BSE-tested beef: An application of mixed error-component logit model. Proceedings of the Agricultural and Applied Economics Association Annual Meeting.

[32] Dang H.D., Pham T.T., Tran G.T., Thi H.A.D., Thi T.M.N. (2020). Vietnamese consumers’ preferences for traceable food and safety attributes: The case of water spinach. J. Asian Bus. Econ. Stud..

[33] Ortega D.L., Wang H.H., Olynk N.J., Wu L., Bai J. (2011). Chinese consumers’ demand for food safety attributes: A push for government and industry regulations. Am. J. Agric. Econ..

[34] Liu R., Gao Z., Nayga R.M., Snell H.A., Ma H. (2019). Consumers’ valuation for food traceability in China: Does trust matter?. Food Policy.

[35] Liu R., Wang J., Liang F., Nian Y., Ma H. (2022). What we can learn from the interactions of food traceable attributes? A case study of Fuji apple products in China. Appl. Econ..

[36] Becker T. (2000). Consumer perception of fresh meat quality: A framework for analysis. Br. Food J..

[37] Swait J., Adamowicz W.L. (1996). The Effect of Choice Environment and Task Demands on Consumer Behavior: Discriminating between Contribution and Confusion.

[38] Louviere J.J., Hensher D.A., Swait J.D. (2000). Stated Choice Methods Analysis and Applications.

[39] Burton M., Rigby D. (2012). The self-selection of complexity in choice experiments. Am. J. Agric. Econ..

[40] DeSarbo W.S., Lehmann D.R., Hollman F.G. (2004). Modeling dynamic effects in repeated-measures experiments involving preference/choice: An illustration involving stated preference analysis. Appl. Psychol. Meas..

[41] Day B., Bateman I.J., Carson R.T., Dupont D., Louviere J.J., Morimoto S., Scarpa R., Wang P. (2012). Ordering effects and choice set awareness in repeat-response stated preference studies. J. Environ. Econ. Manag..

[42] Street D.J., Burgess L. (2007). The Construction of Optimal Stated Choice Experiments: Theory and Methods.

[43] Farrell J., Rabin M. (1996). Cheap talk. J. Econ. Perspect..

[44] Lusk J.L. (2003). Effects of cheap talk on consumer willingness-to-pay for golden rice. Am. J. Agric. Econ..

[45] Murphy J.J., Stevens T., Weatherhead D. (2005). Is cheap talk effective at eliminating hypothetical bias in a provision point mechanism?. Environ. Resour. Econ..

[46] Silva A., Nayga R.M., Campbell B.L., Park J.L. (2011). Revisiting cheap talk with new evidence from a field experiment. J. Agric. Resour. Econ..

[47] McFadden D., Zarembka P. (1974). Conditional logit analysis of qualitative choice behavior. Frontiers in Econometrics.

[48] Train K.E. (2003). Discrete Choice Methods with Simulation.

[49] McFadden D., Train K. (2000). Mixed MNL models of discrete response. J. Appl. Econom..

[50] Greene W.H., Hensher D.A. (2013). Revealing additional dimensions of preference heterogeneity in a latent class mixed multinomial logit model. Appl. Econ..

[51] Boxall P.C., Adamowicz W.L. (2002). Understanding heterogeneous preferences in random utility models: A latent class approach. Environ. Resour. Econ..

[52] Krinsky I., Robb A.L. (1986). On approximating the statistical properties of elasticities. Rev. Econ. Stat..

[53] Ben-Akiva M., Swait J. (1986). The Akaike likelihood ratio index. Transp. Sci..

[54] Roeder K., Lynch K.G., Nagin D.S. (1999). Modeling uncertainty in latent class membership: A case study in criminology. J. Am. Stat. Assoc..

[55] Thiene M., Scarpa R., Longo A., Hutchinson W.G. (2018). Types of front of pack food labels: Do obese consumers care? Evidence from Northern Ireland. Food Policy.

[56] Wu L., Gong X., Qin S., Chen X., Zhu D. (2017). Consumer preferences for pork attributes related to traceability, information certification, and origin labeling: Based on China’s Jiangsu province. Agribusiness.

[57] Garaus M., Treiblmaier H. (2021). The influence of blockchain-based food traceability on retailer choice: The mediating role of trust. Food Control.

[58] Verbeke W., Roosen J. (2009). Market differentiation potential of country-of-origin, quality and traceability labeling. Estey Cent. J. Int. Law Trade Policy.

[59] Wongprawmas R., Canavari M. (2017). Consumers’ willingness-to-pay for food safety labels in an emerging market: The case of fresh produce in Thailand. Food Policy.

[60] El Benni N., Stolz H., Home R., Kendall H., Kuznesof S., Clark B., Dean M., Brereton P., Frewer L.J., Chan M.-Y. (2019). Product attributes and consumer attitudes affecting the preferences for infant milk formula in China–A latent class approach. Food Qual. Prefer..

[61] Albersmeier F., Holger S., Achim S. (2010). System dynamics in food quality certifications: Development of an audit integrity system. Int. J. Food Syst. Dyn..

[62] Hatanaka M., Bain C., Busch L. (2005). Third-party certification in the global agrifood system. Food Policy.

[63] Lusk J.L., Tonsor G.T., Schroeder T.C., Hayes D.J. (2018). Effect of government quality grade labels on consumer demand for pork chops in the short and long run. Food Policy.

[64] Carter D.P., Cachelin A. (2018). The consumer costs of food certification: A pilot study and research opportunities. J. Consum. Aff..

[65] Chen M., Yin S., Xu Y., Chen Y. (2015). Consumers’ willingness to pay for tomatoes carrying different organic labels. Br. Food J..

[66] Brach S., Walsh G., Shaw D. (2018). Sustainable consumption and third-party certification labels: Consumers’ perceptions and reactions. Eur. Manag. J..

[67] Gao Z., Yu X., Li C., McFadden B.R. (2019). The interaction between country of origin and genetically modified orange juice in urban China. Food Qual. Prefer..

[68] Kerr W.A. (2006). Enjoying a good port with a clear conscience: Geographic indicators, rent seeking and development. Estey Cent. J. Int. Law Trade Policy.

[69] Darby K., Batte M.T., Ernst S., Roe B. (2008). Decomposing local: A conjoint analysis of locally produced foods. Am. J. Agric. Econ..

[70] Ortega D.L., Lin W., Ward P.S. (2021). Consumer acceptance of gene-edited food products in China. Food Qual. Prefer..

[71] Sánchez M., Beriain M.J., Carr T.R. (2012). Socio-economic factors affecting consumer behaviour for United States and Spanish beef under different information scenarios. Food Qual. Prefer..

[72] Joya K., Ramli N.N., Shamsudin M.N., Kamarulzaman N.H. (2021). Consumers’ willingness to pay for food safety attributes of tomato. Br. Food J..

[73] Gellynck X., Verbeke W. (2001). Consumer perception of traceability in the meat chain. Ger. J. Agric. Econ..

[74] Ward R.A., Bailey D.V., Jensen R.T. (2005). An American BSE crisis: Has it affected the value of traceability and country-of-origin certifications for US and Canadian beef?. Int. Food Agribus. Manag. Rev..

[75] Tran D., Broeckhoven I., Hung Y., My N.H.D., De Steur H., Verbeke W. (2022). Willingness to pay for food labelling schemes in Vietnam: A choice experiment on water spinach. Foods.

[76] Cox D.F. (1967). The sorting rule model of the consumer product evaluation process. Risk Taking and Information Handling in Consumer Behaviour.

[77] Grunert K.G. (2005). Food quality and safety: Consumer perception and demand. Eur. Rev. Agric. Econ..

[78] Halawany R., Bauer C., Giraud G. Consumers’ acceptability and rejection of food traceability systems, a French-German cross-comparison. Proceedings of the International European Forum on Innovation and System Dynamics in Food Networks Officially Endorsed by the European Association of Agricultural Economists (EAAE).

[79] Golan E.H., Krissoff B., Kuchler F., Calvin L., Nelson K.E., Price G.K. (2004). Traceability in the U.S. Food Supply: Economic Theory and Industry Studies.

